# Nitrogen Substitutions Aggregation and Clustering in Diamonds as Revealed by High-Field Electron Paramagnetic Resonance

**DOI:** 10.1021/jacs.3c06739

**Published:** 2023-12-19

**Authors:** Orit Nir-Arad, David H. Shlomi, Nurit Manukovsky, Eyal Laster, Ilia Kaminker

**Affiliations:** School of Chemistry, Faculty of Exact Sciences, Tel Aviv University, Tel Aviv 6997801, Israel

## Abstract

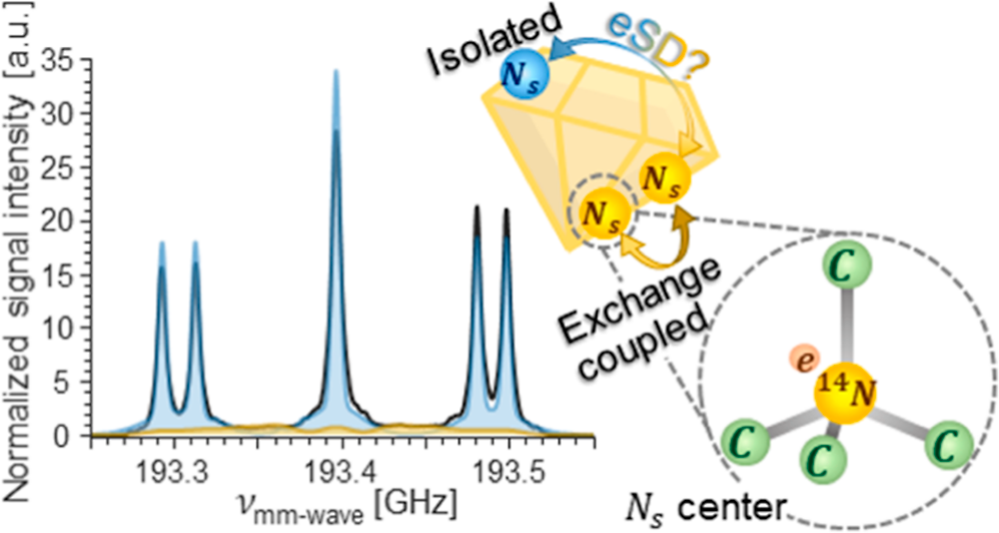

Diamonds have been shown to be an excellent platform for quantum computing and quantum sensing applications. These applications are enabled by the presence of defects in the lattice, which are also known as color centers. The most common nitrogen-based defect in synthetic diamonds is the paramagnetic nitrogen substitution (P1) center. While the majority of quantum applications rely on nitrogen-vacancy (NV) centers, the properties of the latter are heavily influenced by the presence and the spatial distribution of the P1 centers. Hence, understanding the spatial distribution and mutual interactions of P1 centers is crucial for the successful development of diamond-based quantum devices. Unlike NV centers, P1 centers do not have a spin-dependent optical signature, and their spin-related properties, therefore, have to be detected and characterized using magnetic resonance methods. We show that using high-field (6.9 and 13.8 T) pulsed electron paramagnetic resonance (EPR) and dynamic nuclear polarization (DNP) experiments, we can distinguish and quantify three distinct populations of P1 centers: isolated P1 centers, weakly interacting ones, and exchange-coupled ones that are clustered together. While such clustering was suggested before, these clusters were never detected directly and unambiguously. Moreover, by using electron–electron double resonance (ELDOR) pump–probe experiments, we demonstrate that the latter clustered population does not exist in isolation but coexists with the more weakly interacting P1 centers throughout the diamond lattice. Its presence thus strongly affects the quantum properties of the diamond. We also show that the existence of this population can explain recent hyperpolarization results in type Ib high-pressure, high-temperature (HPHT) diamonds. We propose a combination of high-field pulsed EPR, ELDOR, and DNP as a tool for probing the aggregation state and interactions among different populations of nitrogen substitution centers.

## Introduction

Nitrogen impurities frequently occur in both natural and lab-grown diamonds, due to the presence of nitrogen in growth environments and its ease of incorporation into the diamond lattice. Extensive studies focused on nitrogen-based defects^1^ as they significantly affect the properties of the diamond, from coloration^2^ to quantum-level changes such as spin relaxation.^3^ Thus, a great deal of effort is put into controlling the concentration, spatial distribution, and specific types of nitrogen-based defects in diamonds—whether during lab growth^4^ or with post-treatments applicable to natural diamonds as well.^5^

The simplest of nitrogen-based defects is the substitutional nitrogen atom, known as the C-center, N_S_ defect, or P1 center in EPR literature.^1^ When a substitutional nitrogen atom forms a nearest neighbor pair with an adjacent vacancy in the lattice, it can form the negatively charged NV center.^2^ NV centers have received the most attention among the nitrogen-based defects in diamonds due to the combination of their long spin coherence times at room temperature and their optical properties, which enable optical polarization and optical readout of the spin state.^6^ This unique set of properties makes the NV center a promising platform for a variety of novel technologies, such as quantum computing,^7−9^ subpicotesla magnetometry,^10−12^ and microwave amplification.^13^ For some of these applications, a high concentration of NV centers is beneficial,^14^ which has led to the development of methods enriching diamonds with NV centers, to the point where some are commercially available (For example, by Thorlabs).^15^ The precursor for the fabrication of NV centers is the substitutional nitrogen, which is converted to an NV center with at most 25% efficiency.^16^ Thus, NV-enriched diamonds necessarily have a high concentration of P1 centers. These P1 centers have a major impact on the magnetic and spin properties of the NV centers,^3^ highlighting the importance of understanding the spatial distribution of the P1 centers in the diamond lattice.

P1 centers are spin  paramagnetic centers with long coherence times at room temperature,^17^ and have been studied by EPR spectroscopy for decades.^18−20^ They can serve as a very efficient source of ^13^C hyperpolarization in dynamic nuclear polarization (DNP) experiments. Recently, a detailed analysis of DNP mechanisms in a powder of HPHT microdiamonds was carried out by decomposing the DNP spectra acquired at 3.3 T.^21,22^ One of the most intriguing outcomes of this analysis was the significant DNP enhancement on-resonance with the P1 EPR transitions. Such on-resonance DNP is either due to the Overhauser effect (OE)^23^ or due to the recently proposed truncated cross-effect (tCE).^24^ The OE mechanism requires an imbalance between the zero-quantum (ZQ) and double-quantum (DQ) relaxation rates. While common in liquids where the molecular motion results in the spectral density leading to the ZQ–DQ imbalance, in insulating solids OE was observed only for mixed-valence organic radicals.^25−27^ Diamond is an exemplary insulating solid with highly localized defects; therefore, it is not clear what kind of motion will result in the spectral density that will account for the ZQ–DQ imbalance required for OE. On the other hand, the tCE mechanism does not rely on dynamics but instead requires (i) the presence of fast-relaxing paramagnetic species and (ii) these species must interact with the P1 centers. While there is a plethora of paramagnetic impurities known in diamonds, their concentrations in type Ib HPHT diamonds are too low to play a significant role in DNP.^28^ It is therefore unclear which species give rise to this effect. Shimon *et al.* hypothesized the presence of P1 center clusters,^21,22^ but a proof is yet to be provided. The existence of such clusters has also been proposed by Nunn *et al.* based on continuous-wave (CW) EPR power saturation.^29^ Moreover, the clustering of nitrogen defects is a well-known process in the transformation of diamonds from type Ib to type IaA.^30,31^

In this article, using a combination of high-field pulsed EPR and DNP, we were able to identify and quantify the presence of a previously unaccounted-for population of exchange-coupled P1 centers, which is assigned to P1 center clusters. It manifests itself as a broad signal underneath the characteristic P1 center EPR spectrum and is thus hard to detect and identify by using conventional low-field CW EPR techniques. This population is distinct from the exchange-coupled P1 pairs that give rise to an EPR signal with resolved sharp lines in between the ^14^N hyperfine lines in high-power CW EPR experiments.^32−35^

This paper is organized as follows. First, we present ^13^C DNP spectra of type Ib single crystal diamond at 6.9 and 13.8 T. Similar to Shimon *et al.*,^21^ we observed a strong enhancement on-resonance with the EPR transitions, characteristic of the tCE mechanism and thus suggestive of the presence of P1 clusters. Second, we present high-field pulsed EPR spectra together with their simulations, allowing us to directly identify and quantify these clusters. Finally, we use high–field electron–electron double resonance (ELDOR) experiments to demonstrate that the clustered population interacts with the isolated P1 centers, thus fulfilling both conditions for tCE. This finding shows that the exchange-coupled P1 center population provides an efficient pathway for electron–electron spectral diffusion (eSD), which suggests the presence of a new spin-diffusion-based decoherence/relaxation mechanism previously unaccounted for in diamonds. This finding also means that the exchange-coupled P1 centers and the isolated ones are close enough to each other to have a noticeable mutual interaction rather than being in separate parts of the crystal. Our work, therefore, reveals an important property of nitrogen-based defects, which must be considered when designing diamonds tailored for novel applications. Additionally, our work establishes the high-field dual EPR/DNP approach as an informative probe for the detection of strongly interacting, clustered paramagnetic centers beyond what is possible with other methods. Similar conclusions were reached by the group of Han and co-workers.^36^

## Results

**Figure 1:**
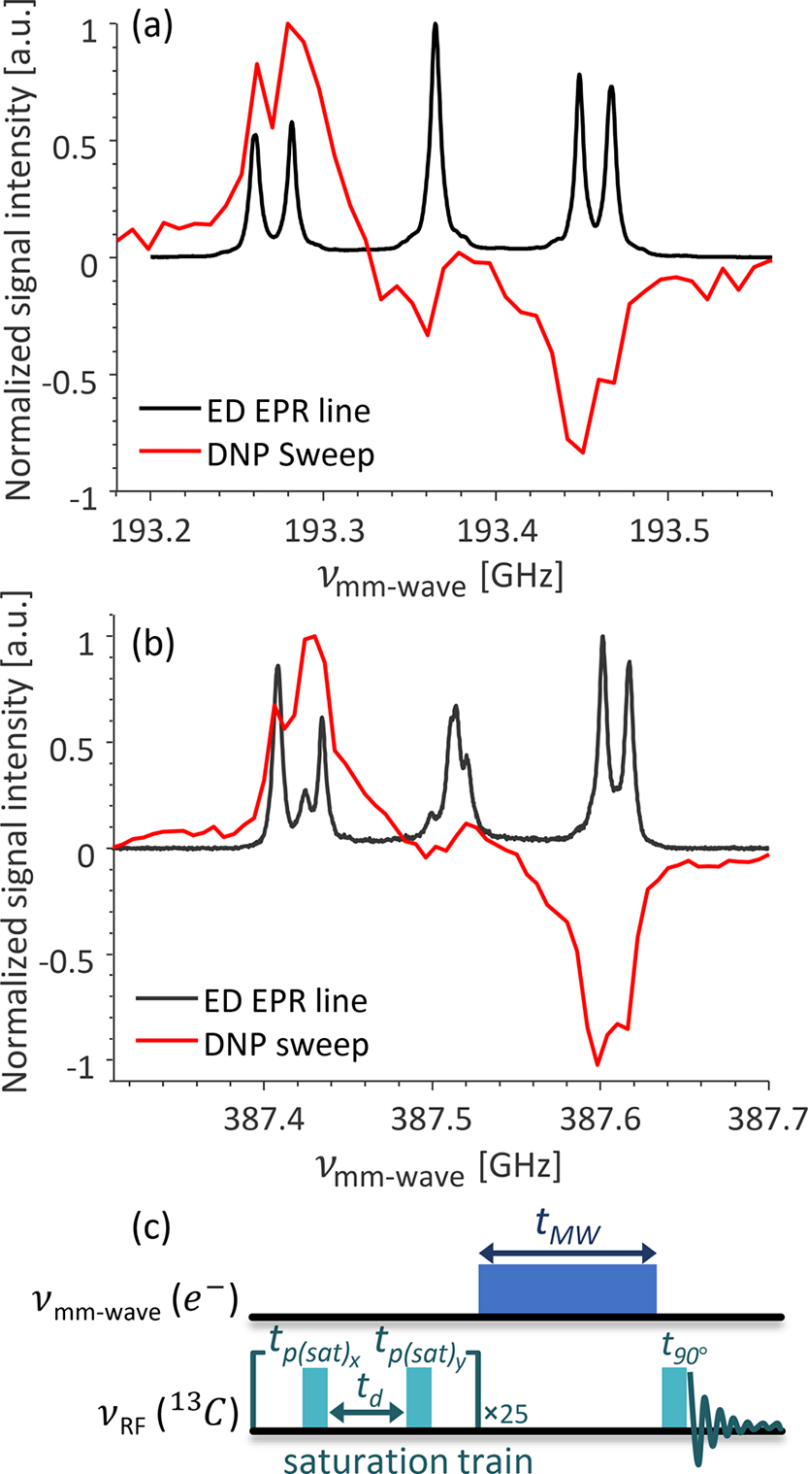
Overlays of ED-EPR spectrum and CW ^13^C DNP sweep of P1 centers in diamond sample A, acquired at (a) 6.9 and (b) 13.8 T. (c) DNP pulse sequence.

Based on previous findings at lower magnetic fields,^22,23^ we wished to examine the efficiency of ^13^C hyperpolarization at 6.9 and 13.8 T and investigate which mechanisms are involved. In particular, we wanted to confirm that the strong on-resonance DNP effect assigned to OE/tCE at 3.3 T is still present at higher magnetic fields. Overlays of the ^13^C DNP and echo-detected (ED) EPR spectra of type Ib HPHT single crystal diamond recorded at 6.9 and 13.8 T are shown in Figure 1a,b, respectively. This sample will be termed diamond A in the rest of the text. The DNP sweep shows the bulk ^13^C nuclear magnetic resonance (NMR) signal intensity after a few minutes of irradiation at a designated frequency (the pulse sequence is shown in Figure 1c). The overall DNP line shape is similar in both fields, with characteristic positive and negative lobes. Clearly, the DNP is the strongest on-resonance with the resolved EPR signals on the left (*m*_I(14N)_ = −1) and right (*m*_I(14N)_ = +1) sides of the EPR spectra, respectively. While the detailed analysis of the DNP spectra is beyond the scope of this article and will be published elsewhere, such on-resonance enhancement was previously assigned to the tCE mechanism.^24^ Other DNP mechanisms are excluded based on the following arguments: solid effect (SE) does not result in on-resonance enhancement. CE requires a pair of electron spins with a frequency separation of ν_13C_. For the 6.9 T spectrum, the frequency separation between the *m*_I_ = ± 1 side and *m*_I_ = 0 central lines is 84 and 104 MHz which exceeds ν_13C_ = 73 MHz. For the 13.8 T spectrum, the ν_13C_ = 146 MHz is larger than the frequency separation between the *m*_I_ = ± 1 and *m*_I_ = 0 and smaller than the 167 MHz separation between the *m*_I_ = +1 and *m*_I_ = −1 lines. The OE could in principle account for the observed effects, but it is hard to envision the dynamics required for the spectral density that will lead to the ZQ–DQ relaxation rate imbalance required for the OE. The tCE DNP mechanism would require the presence of electron spins around the center of the EPR spectrum. To fully understand the DNP results and to uncover and characterize the electron spin population present in the diamond we turned to high-field EPR.

**Figure 2:**
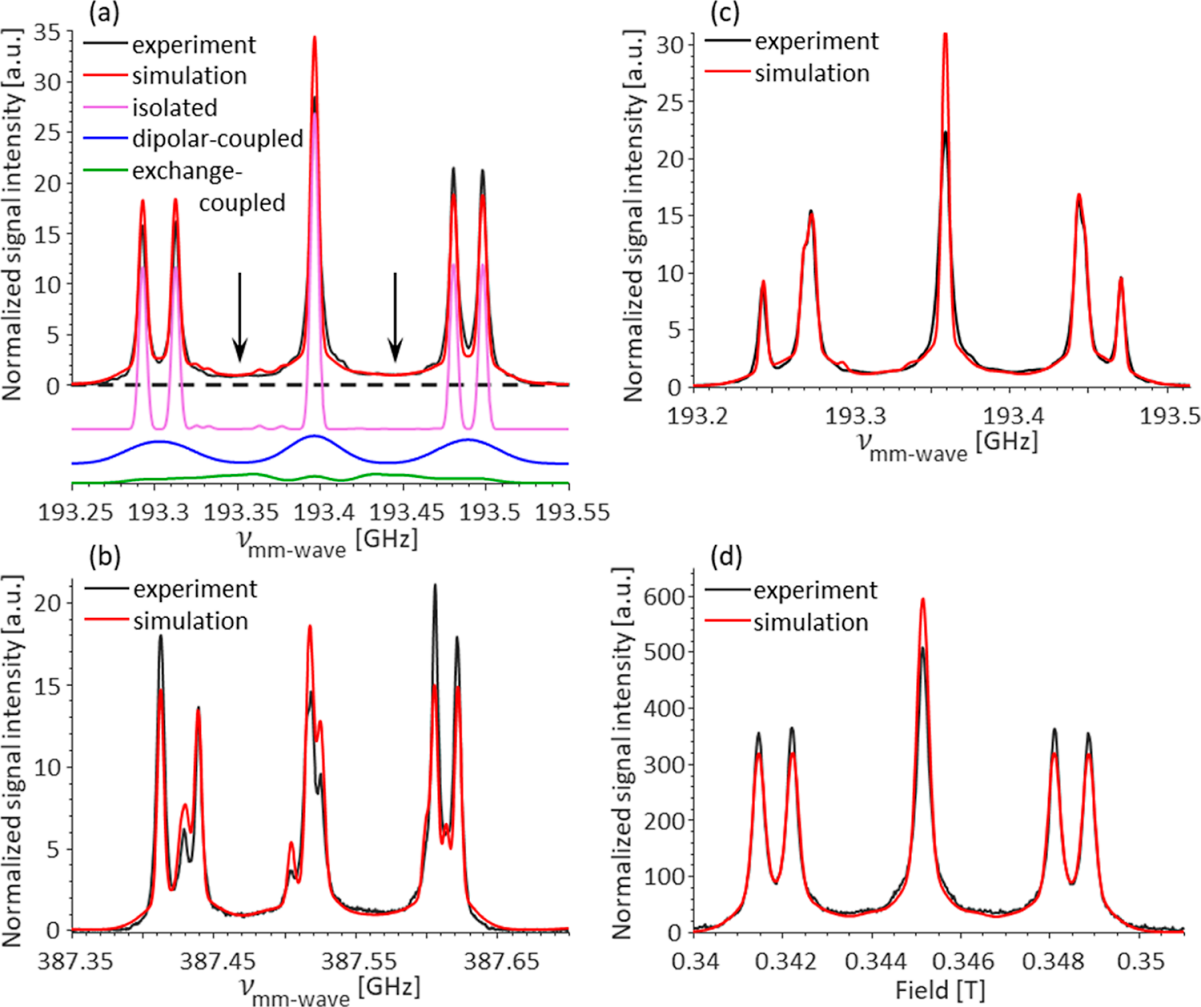
Overlay of the experimental and simulated ED–EPR spectra of P1 centers in HPHT diamonds. (a,b) show the spectra for diamond sample A acquired at 6.9 and 13.8 T, respectively. (a) Also shows a decomposition of the simulated spectrum into individual contributions and arrows pointing to the nonzero signal intensity between the resolved lines of the P1 centers. (c,d) show the spectra for diamond sample B acquired at 6.9 and 0.34 T, respectively.

We have thus proceeded to analyze the high-field EPR spectra of type Ib diamonds in more detail. The echo-detected frequency stepped EPR spectrum of diamond A is shown in Figure 2a in black. The main features visible in the EPR spectrum are the five resolved lines characteristic of the P1 centers in single-crystal diamonds. We note that the asymmetry in the experimental frequency-swept EPR spectra stems from variations in output power with frequency and the presence of standing waves in the quasi-optical system. The ED field-swept spectrum acquired at a constant frequency shown in Figure S1 is symmetric, as expected for the P1 diamond spectra. The number of resolved lines and their exact position depend on the crystal orientation in the magnet. The surprising observation is the nonzero signal intensity between the resolved lines of the P1 center EPR spectrum (marked by arrows in Figure 2a). The paramagnetic species underlying those signals are ideal candidates for the tCE DNP mechanism. To gain more understanding of this nonzero signal intensity, the total observed EPR spectrum was simulated using the EasySpin toolbox.^37^ The simulation is colored red in Figure 2a. To reproduce the observed experimental spectrum, at least three components with distinct parameters had to be used. All three components were simulated with the following Hamiltonian, generally given for *n* electron spin—^14^N nuclear spin pairs:
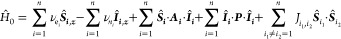
1where  is the electron spin operator with a spin number  and  is the ^14^N spin operator with a spin number 1. The first and second terms of the Hamiltonian are the Zeeman interactions for the electron and ^14^N spins respectively with the external magnetic field, the third is the hyperfine interaction between an electron spin and a ^14^N spin, the fourth is the quadrupole coupling for ^14^N nuclear spins, and the last term is the exchange interaction between two electron spins in close proximity to each other.

The EPR spectrum simulation separated into three components is presented in Figure 2a. For the first two components, we used a single electron spin coupled to a single ^14^N spin. For the third component, we used two such pairs with an exchange interaction between them, with component ratios of 0.56, 0.31, and 0.13 for the “isolated,” “dipolar-coupled” and “exchange-coupled” components, respectively. These ratios indicate the percentage of each population from the total P1 centers in the diamond. The values for the hyperfine and quadrupole interactions, *A*_⊥_ = 81.3 MHz; *A*_∥_ = 114.0 MHz; *P*_∥_ = −3.97 MHz were taken from the literature.^38^*v*_e_ and *v*_n_ are the Larmor frequencies of the electron and ^14^N nucleus, respectively. The “isolated” component of the simulation used a 4.5 MHz line width with a Gaussian broadening (shown in pink in Figure 2a). This is the EPR spectrum typically observed for the P1 centers. The second “dipolar-coupled” component had the same parameters as the first one but with a significantly increased line width of 30 MHz (shown in blue in Figure 2a). The increased line width is assigned to the dipolar interaction, though it was not included explicitly to simplify the simulations. This component represents the P1 centers with a dipolar interaction of up to 47 MHz with each other, estimated from the line width at the bottom of the spectrum. Such a dipolar interaction corresponds to a population with an interspin distance of 1–2.5 nm. The third “exchange-coupled” component (shown in green in Figure 2a) had the same spin Hamiltonian parameters with an addition of the exchange coupling. The line shape is not very sensitive to the exact values of the exchange interaction constant, and *J* = 110–200 MHz gives a satisfactory fit to the experimental data. This third component, which accounts for 13% of the total P1 center population, corresponds to two or more strongly interacting P1 centers, with sub 1 nm interspin distances. This is the population responsible for the nonzero signal in between the EPR signals of the isolated P1 centers’ EPR spectra.

Other possibilities for the nonzero signal between the resolved lines were ruled out based on: (i) the spectrum is centered around the same *g*-factor as the P1 center spectrum, which at the high field of 6.9 T excludes other paramagnetic centers, and (b) we do not observe a symmetric broadening of the P1 center EPR spectrum that could be assigned to the dipolar interaction or *T*_2_ relaxation, but rather the spectrum collapses toward the center which can only be the result of exchange interaction. The exchange-broadened component was not accounted for previously and we suggest that it is the result of several exchange-coupled P1 centers in close proximity. We note that as mentioned in the introduction, this component is distinct from the one observed with high microwave power in low-field CW EPR, as seen in Figure S2 in the Supporting Information, for both diamonds studied in this work. The latter requires ≫1 GHz exchange coupling constant to simulate the results and was assigned to P1 centers pairs.^32^ A similar conclusion on the presence of the exchange-coupled clusters was reached based on the increase in the Rabi nuation frequency for the signals in between the resolved P1 centers in microdiamond powder samples.^36^

Knowledge about the coexistence of three distinct P1 center populations is crucial to the ability to design and characterize diamonds tailored to specific applications. By virtue of these populations having different types and strengths of interspin interactions, their spin properties are bound to be different, and thus also their influence on other defects, *e.g.*, NV centers.

To corroborate these findings, we performed an even higher field EPR experiment at 13.8 T, which to the best of our knowledge is the highest magnetic field at which P1 centers were measured. Notably, the forbidden transitions, that are very weak in 6.9 T spectra, become very pronounced at 13.8 T due to the cancellation condition (ν_14N_ ≈ *A*_eff_/2)^39^ resulting in an unfamiliar-looking spectrum. The spectrum is shown in Figure 2b and was fitted using the same parameters and line widths used to simulate the 6.9 T spectrum. The same three components with the same component ratios for the isolated, dipolar-coupled, and exchanged-coupled components are present in the 13.8 T EPR spectrum as well, confirming our interpretation. The 13.8 T ED EPR experiments are even more sensitive to the g-tensor accuracy, and we could refine the literature values for the *g*-tensor to *g*_⊥_ = 2.00220 ± 0.00001; *g*_∥_ = 2.00218 ± 0.00001. The procedure is detailed in the Supporting Information. While these values are lower than the originally reported one of *g*_iso_ = 2.0024,^18,40,41^ they are consistent with later reports.^28,42,43^

Further confirmation of the validity of our interpretation is that we could simulate an EPR spectrum of a second sample of Ib HPHT single crystal diamond, termed diamond B in the rest of the text. The 6.9 T EPR spectrum and the corresponding simulation are shown in Figure 2c. We were able to simulate this spectrum with the same three components using the same parameters, exchange interaction constant, and line widths, except for the isolated line width that changed slightly between the two, as for the first diamond while changing only the ratio between the components to 0.47, 0.38, and 0.15 for the isolated, dipolar-coupled, and exchanged-coupled components, respectively. This result demonstrates that while all three P1 center populations are present in both studied diamonds, their relative amounts and distributions are different. Similarly, the 13.8 T spectrum of diamond B could be simulated using the same three components as well (Figure S3).

To further test our interpretation, we measured another pulsed EPR spectrum of diamond B, this time acquired by using a commercial X-band (0.35 T) instrument (Figure 2d). While it is possible that relaxation times change drastically at different magnetic field strengths, which would affect the ratio between the three components in our spectra, this was not the case. We were able to simulate the X-band spectrum using the same three components with the same component ratios and line widths, highlighting the ability of pulsed EPR to differentiate and quantify these three P1 center populations. It is important to note that while the X-band spectrum clearly shows the presence of the nonzero signal intensity between the resolved EPR lines, insufficient *g*-factor resolution makes it on its own incapable of distinguishing this population from other EPR defects with broad EPR lines, *e.g.*, dangling carbon bonds,^44,45^ and therefore quantifying it. Interestingly, while the exchange-broadened P1 center population seems to be ubiquitous in type Ib diamonds, it is not easily observed by conventional CW EPR (Figure S2a,b).

Long coherence times are crucial for many modern diamond applications, such as quantum computing, quantum memory, and quantum sensing. The different P1 center populations are likely to have different influences on the coherence times of other defects. We thus proceeded to characterize the relaxation times for the isolated and exchange-broadened components of the EPR spectrum.

**Figure 3:**
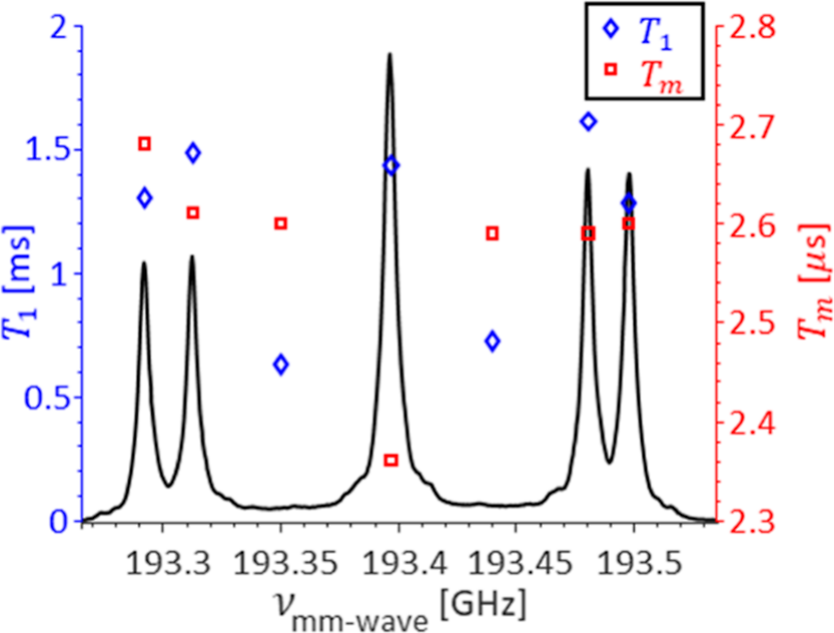
*T*_1_ and *T*_m_ relaxation times measured across the EPR spectrum of diamond A.

The spin–lattice relaxation time *T*_1_ was measured using a saturation recovery sequence, and the phase memory time *T*_m_ using a two-pulse echo decay sequence. The *T*_1_ and *T*_m_ relaxation times measured at different positions across the EPR spectrum of diamond A at 6.9 T are shown in Figure 3 and summarized in Table S1 in the Supporting Information. We find that *T*_1_ is shorter for the exchange-coupled population compared to that for the isolated P1 centers. The shortening of *T*_1_ due to an increase in exchange interaction was observed before in solid solutions of DPPH in polystyrene^46^ and in amorphous hydrogenated carbon.^47^ Surprisingly, we do not find significant differences in *T*_m_ relaxation between the *m*_I_ = ± 1 of the isolated component and the exchange-broadened components, with all the data falling into the 2.59–2.68 μs range, the *T*_m_ for the central *m*_I_ = 0 peak being shorter and equals 2.36 μs. The similarity between these values suggests that the same *T*_m_ relaxation mechanism dominates for all of the species in this sample. Similar results were reported for synthetic type Ib diamonds at lower magnetic fields.^48^ The large differences in *T*_1_ relaxation between the exchange-coupled and isolated components further confirm that the exchange-coupled population of P1 centers is distinct from those that interact with their neighbors only via the dipolar coupling.

**Figure 4:**
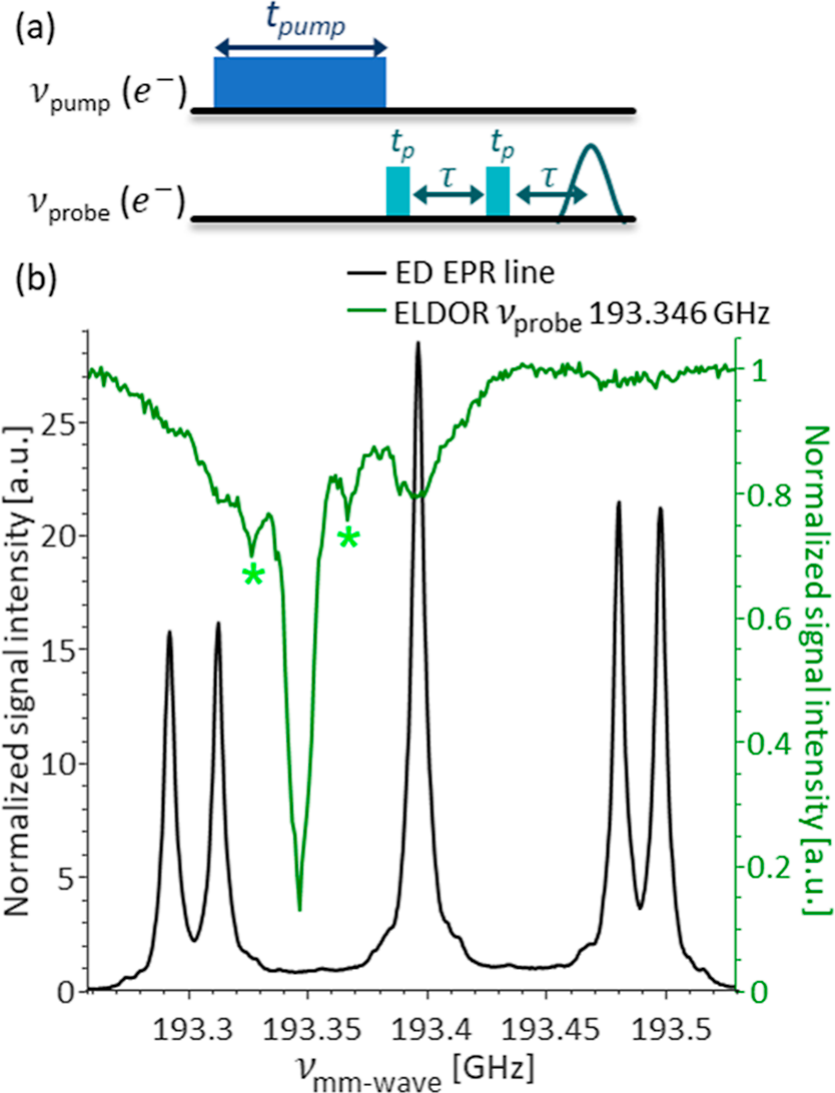
(a) Pulse sequence for the ELDOR experiment. (b) ELDOR spectrum of P1 centers in diamond A overlaid with the experimental EPR line. The positions of the weakly coupled ^14^N peaks are marked with asterisks.

We then proceeded to investigate the interactions between the isolated and exchange-coupled P1 centers. The presence or absence of such interactions will indicate whether each of the three populations of P1 centers resides in a separate region in the diamond lattice or they coexist side by side on the nm scale. To this end, we used the ELDOR experiment, which probes the interaction between different parts of the EPR spectrum. It is a pump–probe experiment where the influence of a long pulse at *v*_pump_ frequency is probed at a different *v*_probe_ frequency using the echo sequence as shown in Figure 4a. During the experiment, *v*_probe_ remains constant and *v*_pump_ is stepped. The ELDOR experiment allowed us to observe the interaction between the exchange-coupled and isolated P1 center populations. Figure 4b shows the ELDOR spectrum with *v*_probe_ = 193.346 GHz. This frequency was chosen to avoid any forbidden EPR transitions of the isolated P1 center population in order to probe only the interactions involving the exchange-coupled population.

The overall shape of the ELDOR spectrum is consistent with a spin system with an eSD. Spectral diffusion manifests itself as propagation of excitation throughout the inhomogeneously broadened EPR line,^49^ and scales inversely with the square of the frequency separation,^50^ resulting in stronger ELDOR signals when the frequency separation between the *v*_pump_ and *v*_probe_ is small, and a decrease in signal intensity with an increase in the frequency separation. The ELDOR signal intensity also depends on the local concentration of the paramagnetic species, with an increase in the interspin distance resulting in a less pronounced spectral diffusion.

As expected, the strongest signal is for *v*_pump_ = *v*_probe_, and we observe weaker ELDOR signals that coincide with all EPR signals of the isolated P1 centers population, thus confirming the presence of eSD between the two populations. The two signals at 193.326 and 193.366 GHz (marked with bright green asterisks in the figure) are assigned to weakly interacting remote ^14^N nuclei with small hyperfine interactions. These are common in ELDOR spectra and are the result of the excitation of the DQ and ZQ transitions of weakly coupled ^14^N and are not related to the eSD. An additional ELDOR spectrum acquired at 193.450 GHz corroborating our conclusions is shown in the Supporting Information in Figure S4.

The eSD was shown to have a profound influence on the DNP mechanisms in nitroxide radicals^51^ and we hypothesize it to be similarly important for type Ib diamonds. Using the ELDOR spectrum, we have shown that eSD on the millisecond time scale relevant for the DNP experiments exists between the isolated and exchange-coupled populations.

## Conclusions

The DNP spectra of type Ib diamonds acquired at 6.9 and 13.8 T suggested the presence of a paramagnetic population that does not belong to the characteristic P1 center EPR spectrum. Using pulsed EPR spectra, recorded at record-high fields of 6.9 and 13.8 T, we directly observed a previously unaccounted-for population of P1 centers that can explain the DNP line shape. The only explanation for the observed EPR spectra is the presence of spatially close P1 centers with an exchange coupling on the order of hundreds of MHz between them. This population appears to be ubiquitous among synthetic type Ib diamonds, amounts to 10–15% of all P1 centers, and has distinct spin–lattice (*T*_1_) relaxation times compared to the isolated P1 centers. Using ELDOR experiments, we have shown that the isolated and exchange-coupled populations coexist throughout the diamond lattice and interact with each other via the eSD mechanism. We suggest that the exchange-coupled population will similarly influence the NV center ensemble and thus has to be taken into account in synthetic diamond design.

## Materials and Methods

Diamond A is a 3.2 × 3.2 × 1.1 mm HPHT diamond single crystal with a uniform yellow color polished to the [100] face. It was purchased from Element 6. Diamond A was placed close to the [111] orientation for the measurement to simplify the spectrum. According to the manufacturer, diamond A has a boron concentration below 0.1 ppm and a nitrogen concentration below 200 ppm. The P1 center concentration is ∼20 ppm.

Diamond B is an HPHT crystal of irregular shape with approximate dimensions of 3.3 × 2 × 0.3 mm and a uniform pink color. It has a nitrogen concentration of ∼100 ppm and a P1 center concentration of 30–45 ppm.

The spin counting was performed on a CW X-band Bruker Elexsys E500 spectrometer.

High field measurements were performed on a home-built spectrometer at 6.9 and 13.8 T at room temperature. The spectrometer uses a home-built pulse forming unit and an amplifier multiplier chain (AMC) with an output power of 375–450 and 90–100 mW for the 193 and 386 GHz range, respectively. A phase-sensitive EPR detection is performed by using an induction mode quasi-optical setup and a superheterodyne scheme. The details of the spectrometer design are published elsewhere.

The CW ^13^C DNP spectra were acquired using the pulse sequence shown in Figure 1c. The continuous wave mm-wave irradiation was applied for time *t*_MW_ of 120 and 300 s, *t*_90_ was 12 and 14.5 μs, and the total experiment time was 17 and 14 h for 6.9 and 13.8 T experiments, respectively. Each experiment started with a saturation train on the carbon channel, consisting of 50 *t*_90_ pulses with alternating phases of 0° and 90° and an interpulse delay *t*_d_ of 30 μs.

The ED frequency stepped EPR spectra acquired at 6.9 and 13.8 T were measured using a *t*_p_ – τ – *t*_p_ – τ – echo sequence. A 16-step phase cycle with , and ϕ_detection_ = ϕ_p1_ – 2ϕ_p2_ was implemented to account for mixer imperfections. The parameters used for each spectrum are summarized in Table 1.

**Table 1:** Experimental Parameters Summary for the High-Field ED Frequency-Stepped EPR Spectra

Figures	2a	2b	2c	S3
Diamond	A	A	B	B
Field [T]	6.9	13.8	6.9	13.8
*t*_p_ [μs]	0.9	1.4	0.5	1
τ [μs]	1.2	0.9	0.5	0.5
Repetition time [ms]	2	0.3	2	2
Averages per point	20	20	50	10

The ED field swept spectrum of diamond A at 6.9 T, in the Supporting Information, was acquired using the same pulse sequence as the frequency stepped ones at a constant frequency of 193.6 GHz and a field sweep rate of 25 μT/s.

Two-pulse echo decay was measured with the same pulse sequence incrementing τ, with *t*_p_ of 0.9 μs, repetition time of 3 ms, and 100 averages per point. The data were fitted using a single exponential decay.

Saturation recovery was performed using a saturation train and the same echo sequence as before by incrementing τ_d_ delay separating the two (Figure S5), [*t*_p(sat)*x*_ – *t*_p(sat)*y*_]_500_ – τ_d_ – *t*_p_ – τ – *t*_p_ – τ – echo with *t*_p(sat)*x*_ = *t*_p(sat)*y*_ = 1 μs, *t*_p_ was 0.9 μs and τ was 0.9 μs and the same 16-step phase cycle for the echo sequence was implemented. Repetition time was 20 ms with 50 averages per point. The data were fitted using a stretched exponential function. The stretching parameter β was 0.87 for the isolated component and 0.68 for the exchange-coupled one.

ELDOR experiments (Figures 4a and S4) were performed with a 50 ms pump pulse and the same echo detection sequence as in the frequency-stepped EPR spectrum with 100 averages per point and a repetition time of 54 ms with a total experiment time of 12 h. The spectra are normalized to the echo intensity with *v*_pump_ off-resonance from the EPR spectrum.

Pulsed X-band EPR measurements were performed on a Bruker Elexsys E580 spectrometer. The ED field swept spectrum was measured using a *t*_90°_ – τ – *t*_180°_ – τ – echo sequence with *t*_90°_ and *t*_180°_ of 60 and 120 ns, respectively and τ of 190 ns, a microwave frequency of 9.68 GHz, repetition time of 5 ms, 30 averages per point, and a 2-step phase cycle.

The CW X-band EPR measurements were performed on a Bruker EMX spectrometer. The CW field-swept spectra in the Supporting Information were measured with a microwave frequency of 9.28 GHz, modulation amplitude of 1 G, low output power measurements using 0.18 mW, and high output power measurements using 4.5 and 22 mW for diamonds A and B, respectively.

The simulations were performed using the MATLAB EasySpin toolbox^37^ using the pepper function for solid-state simulations. The deviation from the [111] orientation of each diamond relative to the main magnetic field was determined first, using a single component simulation of the isolated component, a relative orientation of [45, 54.74, 0]° for the P1 center molecular frame relative to the diamond crystal, and a crystal symmetry of *Fd*-3*m*. Diamond A had an 8.1° deviation, both at 6.9 and 13.8 T, and for diamond B the deviation in Euler angles was [57, −9.8, 0]°, [34, 0.5, 0]°, and [0, 1.7, 0]° for 13.8, 6.9, and 0.34 T, respectively. The 13.8 T simulation of the isolated component also allowed us to determine the g-tensor components. A total of 6 free parameters (the three Euler angles, *g*_∥_, *g*_⊥_ , and line width) were used in the isolated component simulation.

After fixing the parameters for the isolated components, the full 13.8 T spectra were fitted using the three-component simulation with the exchange interaction constant and each component’s line width and relative weight as the only free parameters (a total of 7 free parameters). The lower field spectra were fitted with only the line width or relative weight of each component as the free parameters (a total of 3 free parameters), with the higher-field simulation parameters resulting in the best fit to the experimental data. The isolated component line width was 4.52 and 4.01 MHz for diamonds A and B respectively. At 0.34 T, the diamond B simulation used a line width of 7.23 MHz. The dipolar-coupled component line width was 30 MHz for all spectra simulated. The exchange-coupled component used two electron spins with an exchange coupling of 138 MHz and no dipolar interaction, each electron spin is coupled to a single ^14^N spin. The large line width of 17.5 MHz was used to account for any dipolar-broadening present in addition to the exchange coupling. The exchange-coupled component parameters were the same for all spectra simulated. For diamond, A (B) The relative weight of each component, determined by the relative integral of each component, was 0.56, 0.31, and 0.13 (0.47, 0.38, and 0.15) for the isolated, dipolar-coupled, and exchange-coupled, respectively. Both the experimental spectra and the simulations were normalized to their respective integrals.

## Supporting Information Available

6.9 T field swept ED-EPR of diamond A, 0.34 T CW EPR spectra of diamond A and B, 13.8 T ED-EPR of diamond B, additional ELDOR spectrum, spin–lattice relaxation measurements, and calculation of the *g*-tensor parameters (PDF)
