# Supplementary material for: Nitrogen Substitutions Aggregation and Clustering in Diamonds as Revealed by High-Field Electron Paramagnetic Resonance
Source: J Am Chem Soc. 2023 Dec 19;146(8):5100–7. doi: 10.1021/jacs.3c06739 (PMC10910503; doi:10.1021/jacs.3c06739)
Supplement: Supplementary file 1 — ja3c06739_si_001.pdf [file ja3c06739_si_001.pdf]

Supporting Information for Nitrogen Substitutions Aggregation and Clustering in Diamonds as Revealed by High-Field Electron Paramagnetic Resonance

Orit Nir-Arad[1], David H. Shlomi[1], Nurit Manukovsky[1], Eyal Laster[1], and Ilia Kaminker[*1].

[1]School of Chemistry, Faculty of Exact Sciences, Tel Aviv University, Tel Aviv, 6997801 Israel.
**Email: iliakam@tauex.tau.ac.il*

6.9 T Field Swept ED-EPR of Diamond A

The asymmetry in signal intensity between the left and right sides of the experimental spectrum of diamond A in Figure 2a, as well as the remaining discrepancy in signal intensity between the simulation and the experiment, are due to the dependence of power output on the mm-wave frequency and the presence of standing waves in the quasi-optical system. For accurate signal intensity, we measured an echo-detected (ED) field sweep EPR spectrum at a constant frequency (Figure S1), which indeed shows the expected symmetric EPR spectrum. The ED field-swept EPR spectrum was measured using the same pulse sequence and parameters as the frequency-stepped EPR spectrum.

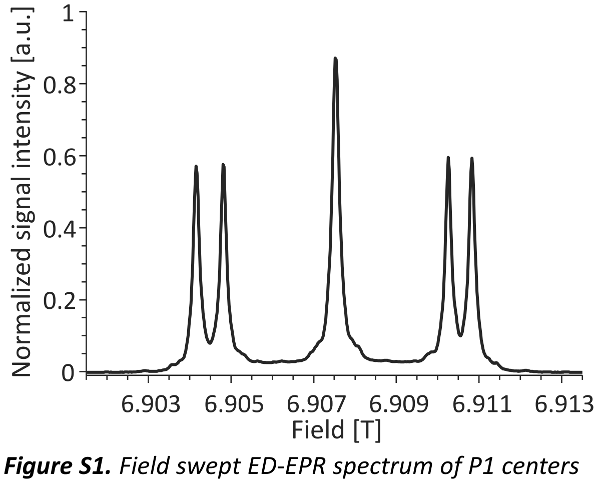

Figure S1. *Field swept ED-EPR spectrum of P1 centers in diamond A acquired at 6.9 T.*

0.34 T CW EPR Spectra

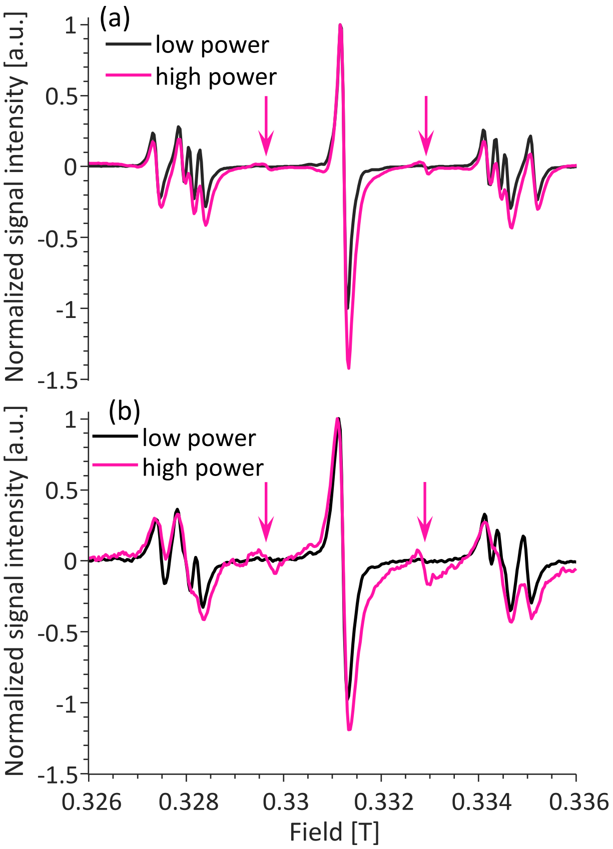

Figure S2. *(a) and (b) show CW field sweep of HPHT diamonds A and B acquired at X-band. The magenta-colored arrows point to the additional transitions between the resolved ^{14}N hyperfine lines, which are visible only with high microwave power.*

In order to confirm that the resolved lines observed at low-field CW EPR experiments between the resolved outer ^{14}N hyperfine lines belong to a different exchange-coupled species than those responsible for the broad signal in pulsed EPR spectra, we recorded CW EPR spectra

at 0.34 T. Sharp, resolved lines appear between the m_I = ±1 and m_I = 0 lines in CW EPR spectra acquired with high microwave power as shown in Figures S2a and b. Therefore both types of exchange-coupled P1 centers exist in Diamond A and Diamond B samples, but the two have very different EPR spectra.

13.8 T ED-EPR of Diamond B

An EPR spectrum of diamond B acquired at 13.8 T overlaid with the corresponding simulation is shown in Figure S3. An additional signal marked with an asterisk belongs to the NV center and is thus not accounted for by the simulation.

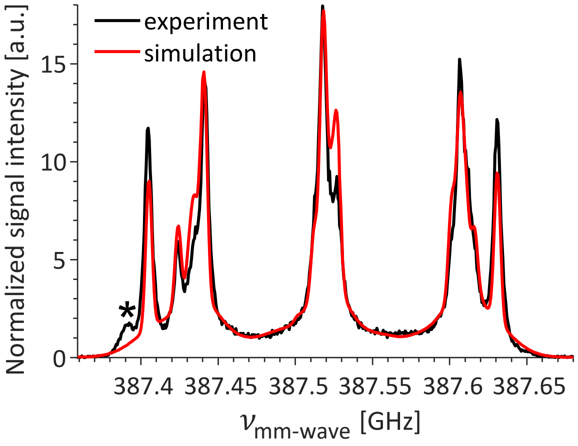

Figure S3. *Overlay of experimental and simulated ED-EPR spectrum of P1 centers in diamond B acquired at 13.8 T. An additional non-P1 peak is marked with an asterisk.*

Additional ELDOR Spectrum

Figure S4 shows ELDOR with ν_{probe} = 193.45 GHz, corresponding to the excitation of the exchange-coupled population on the right side of the EPR spectrum. The spectrum in the figure mirrors the one shown in Figure 4b of the main text and corroborates our conclusions.

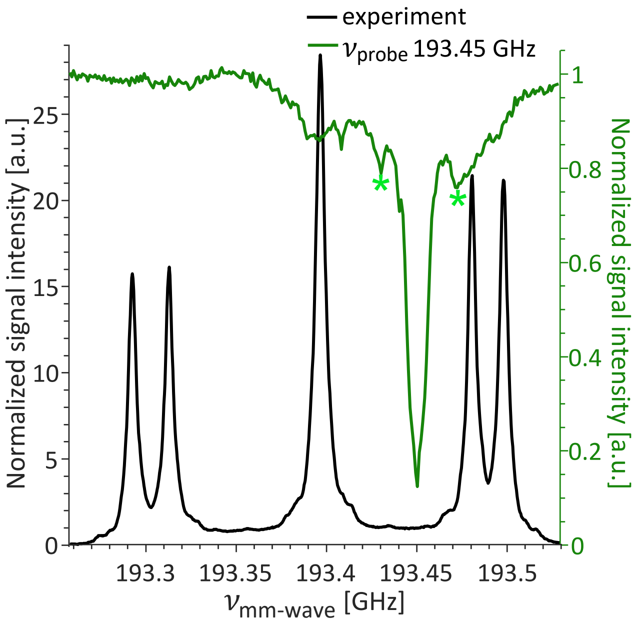

Figure S4. *ELDOR spectrum acquired at 193.450 GHz of P1 centers in diamond A overlaid with the experimental EPR line. The asterisks mark the positions of non-eSD ^{14}N peaks.*

Spin-Lattice Relaxation Measurements

An example of saturation recovery experimental data, measuring spin-lattice relaxation time T_1, for $\nu_{mm-wave}$ = 193.397 GHz is shown in Figure S5. The experiment was recorded using the pulse sequence in the inset with parameters listed in the materials and method section in the main text. The data in Figure S5 was fitted using mono- and stretched-exponential functions showing a clear preference for the latter.

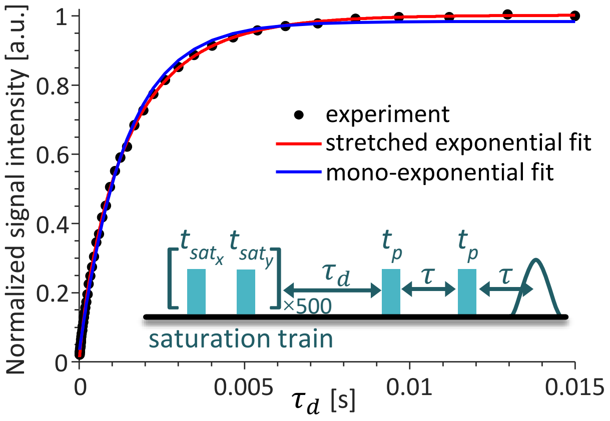

Figure S5. *Saturation recovery of P1 centers in HPHT diamond with $\nu_{mm-wave}$ = 193.397 GHz with mono- and stretched exponential fit.*

The relaxation times measured at the other $\nu_{mm-wave}$ frequencies for diamond A are summarised in Table S1 and Figure 3 in the main text.

Frequency [GHz]	T_1 [ms]	β	T_m [μs]
193.292	1.305	0.87	2.676
193.313	1.484	0.85	2.606
193.35	0.630	0.67	2.596
193.397	1.436	0.86	2.360
193.44	0.722	0.68	2.593
193.481	1.612	0.88	2.594
193.498	1.282	0.88	2.602

Table S1. *Summary of T_1 and T_m relaxation times measured across the EPR spectrum of diamond A, with β as the stretching exponent for the stretched exponential fit of the T_1 relaxation.*

Calculation of the g-tensor

The g-tensor was determined from the simulation of the frequency sweep at 13.8 T. To accurately calculate the magnetic field we used the measured radio frequency (RF) of the diamond NMR signal.

$$(S1)\ \nu_{RF} = \frac{\gamma_{13C}}{2\pi \times 10^6} \cdot B_0$$

With ^{13}C gyromagnetic ratio $\gamma_{13C} = 6.728286 \times 10^7 \frac{rad}{s \cdot T}$.

Since the gyromagnetic ratio is defined for the free atom, we need to account for the chemical shift (CS) of diamond relative to it. For ^{13}C NMR all CS values are reported relative to the reference sample of Tetramethylsilane (TMS) in deuterated chloroform (Me_4Si in $CDCl_3$), thus we began by calculating the CS of TMS relative to the free ^{13}C atom and then accounted for the CS of diamond relative to TMS. We used the literature value for the theoretical Larmor frequency of free ^{13}C atom of 25.1504 MHz, and the experimental frequency of 1% Me_4Si in $CDCl_3$ sample 25.145020 MHz, both at the magnetic field strength where proton NMR signal of TMS equals 100.0 MHz. (All values taken from Bruker NMR properties of selected isotopes table)

$$(S2)\ \frac{(\nu_{TMS} - \nu_{ref})}{\nu_{ref}} \times 10^6$$

$$= \frac{25.145020 - 25.1504}{25.1504} \times 10^6$$

$$= -213.913\ \text{ppm}$$

Diamond CS relative to TMS is known as 33 or 36 ppm[1,2], thus the CS of diamond relative to the free atom is -180.913 or -177.913 ppm which for the 100 MHz proton field give 25.14588 MHz. The B_0 for the ED-EPR spectra was calculated using this value.

Both CS values result in final g-tensor values of $g_\perp$ = 2.00220 ± 0.00001; $g_\parallel$ = 2.00218 ± 0.00001 with the difference in the two CS values manifesting itself as the difference in the g-tensor values of 0.000006-0.000007, which is smaller than the error.

References

(1) Duijvestijn, M. J.; van der Lugt, C.; Smidt, J.; Wind, R. A.; Zilm, K. W.; Staplin, D. C. 13C NMR Spectroscopy in Diamonds Using Dynamic Nuclear Polarization. *Chem. Phys. Lett.* **1983**, *102* (1), 25–28. https://doi.org/10.1016/0009-2614(83)80650-2.

(2) Duncan, T. M. 13C Chemical Shieldings in Solids. *J. Phys. Chem. Ref. Data* **1987**, *16* (1), 125–151. https://doi.org/10.1063/1.555789.
